# Association of waist circumference, body mass index and conicity index with cardiovascular risk factors in postmenopausal women

**DOI:** 10.5830/CVJA-2012-038

**Published:** 2012-09

**Authors:** Shidfar Farzad, Alborzi Fatemeh, Maryam Salehi, Marzieh Nojomi

**Affiliations:** Department of Nutrition, School of Health, Tehran University of Medical Sciences, Tehran, Iran; Department of Community Medicine, Tehran University of Medical Sciences, Tehran, Iran; Department of Community Medicine, Tehran University of Medical Sciences, Tehran, Iran; Department of Community Medicine, Tehran University of Medical Sciences, Tehran, Iran

**Keywords:** body mass index, cardiovascular risk factors, conicity index, waist circumference

## Abstract

**Abstract:**

In menopause, changes in body fat distribution lead to increasing risk of cardiovascular disease and metabolic disorders. The aim of this study was to assess the association of adiposity using the conicity index (CI), body mass index (BMI) and waist circumference (WC) with cardiovascular risk factors (hypertension, diabetes and dyslipidaemia). The sample of this cross-sectional study was collected from June to October 2010 and 165 consecutive menopausal women who had attended the Health and Treatment Centre and Endocrine Research Centre of Firoozgar Hospital in Tehran, Iran were assessed. Age, weight, height, WC, waist–hip ratio (WHR), CI and fat mass were measured. Systolic and diastolic blood pressure (SBP and DBP), fasting blood glucose, insulin, low-density lipoprotein cholesterol (LDL-C), high-density lipoprotein cholesterol (HDL-C) and total cholesterol (TC) levels were also determined. All statistical analyses were performed by SPSS version 17 (SPSS Inc, Chicago, IL, USA).

Results showed that BMI was positively and significantly associated with SBP (*r* = 0.21; *p* = 0.009). WC was positively and significantly correlated with SBP (*r* = 0.26; *p* = 0.02) and DBP (*r* = 0.16; *p* = 0.05). WHR was also significantly and positively associated with SBP (*r* = 0.29; *p* = 0.001). Age and WC were associated with CI quartiles at the 0.05 significance level. The correlation of CI quartiles with SBP and weight were at the 0.01 significance level.

We showed a significant association of WC with SBP and DBP, and that BMI could be an important determining factor of SBP. For assessing the association between CI and cardiovascular risk factors, future studies with larger sample sizes are recommended.

## Abstract

Epidemiological studies have found a progressive increase in the prevalence of cardiovascular risk factors (dyslipidaemia, elevated blood pressure, disturbances in glycaemic control) with increasing body fatness.^1^-^3^ In recent decades, many prospective and cross-sectional studies using anthropometric measures have been undertaken in order to understand the relationship between obesity and cardiovascular risk factors. Various obesity measurements such as body mass index (BMI), waist circumference (WC) and waist-to-hip ratio (WHR) were investigated. However, the best obesity measure to use as a predictor of cardiovascular risk factors remains elusive.^4^-^5^

BMI is the most commonly used and simple measure of body size, especially for estimating the frequency of obesity in large epidemiological studies.^6^ This index cannot however be used for the evaluation of body fat distribution and abdominal fat mass. It has been shown that intra-abdominal fat has a stronger relationship with risk of obesity-related morbidity than with overall adiposity.^7^ Therefore WHR and WC measurements can be used as valid alternatives to BMI for the evaluation of intra-abdominal mass and total fat.^8^

A study by Huang and co-workers showed that WHR and WC measurements were strongly associated with incidence of coronary heart disease, independent of BMI.^9^ However the validity of WHR measurements has been questioned as an indicator of abdominal adipose tissue distribution.^10^

Another index of abdominal adiposity is the conicity index (CI). This has a theoretical range, includes a built-in adjustment of waist circumferences for height and weight, and does not require the hip circumference to assess fat distribution.^11^,^12^

In menopause, changes in body fat distribution lead to increasing risk of cardiovascular and metabolic diseases. Increase in abdominal obesity together with acceleration of the breakdown of lean body mass means there is no significant change in the body weight of postmenopausal women.^7^,^8^

Since the prediction of cardiovascular disease by the presence of risk factors is of such importance, anthropometric indices are seen as useful indicators to achieve this. The aim of this study was to assess the association of adiposity using the conicity index, BMI and WC, with cardiovascular risk factors (hypertension, diabetes and dyslipidaemia).

## Methods

This cross-sectional study was carried out from June to October 2010. Using the non-probability convenience method, 165 consecutive menopausal women who had attended the Health and Treatment Centre and Endocrine Research Centre of Firoozgar Hospital in Tehran, Iran, were invited to participate. Subjects were informed on the objectives of the study. The study was approved by the ethics committee of the Medical School of Iran University of Medical Sciences.

Inclusion criteria for the study were: being naturally menopausal for at least one year, non-smokers, and having a BMI less than 30 kg/m^2^. We excluded women with severe liver, renal and cardiovascular disorders, and those who were on medication. Women on hormone replacement or lipid-reducing therapy, antihypertensive drugs and non-steroidal anti-inflammatory drugs (NSAIDs) were also excluded.

Variables included age, weight, height, WC, WHR, CI and fat mass. Systolic and diastolic blood pressure, and laboratory variables including fasting blood glucose, insulin, low-density lipoprotein cholesterol (LDL-C), high-density lipoprotein cholesterol (HDL-C) and total cholesterol (TC) levels were measured. All participants were asked to complete a three-day dietary-recall questionnaire and a food-frequency questionnaire.

Weight, height, and hip and waist circumference were measured using standard procedures. Weight was measured to the nearest to 0.1 kg, without shoes and wearing light clothing. Height was recorded to the nearest 0.1 cm. WC was measured using a rubber measuring tape, horizontally halfway between the lower border of the rib cage and the iliac crest. Hip circumference (HC) was measured at the widest part over the buttocks. WC and HC were measured to the nearest 0.5 cm. The WHR was calculated by dividing the WC (cm) by the HC (cm). BMI was determined as weight (kg) divided by height (m^2^). The CI was calculated using weight (kg), height (m) and WC (m) as follows: Conicity index = $\frac{waist\;circumreferece\;(m)}{\sqrt{0.109\sqrt{weight}\left(kg\right)}/height\;(m)}$

Fat mass was determined by BIA (Body Stat, UK). Blood pressure was measured after 10 minutes’ rest, with the subjects in a seated position. Systolic and diastolic blood pressure (SBP and DBP, respectively) were measured with two readings at one-minute intervals. The mean was the average of two readings. All measurements were recorded to the nearest 2 mmHg.

Following at least 12 hours’ fast, blood was taken for assessment of total cholesterol (TC), fasting blood glucose (FBS), triglyceride, HDL-C and LDL-C levels. These were determined by enzymatic procedures (Pars Azmon Kit, Tehran, Iran). Plasma insulin level was measured by ELISA (Diaplus Inc. Kit, North York, ON, Canada).

Diabetes was defined as a history of diabetes diagnosed by a physician, taking hypoglycaemic medication, or having a FBS level more than 126 mg/dl.

## Statistical analyses

Descriptive statistics for variables were used with tables, means and standard deviations. The association of WC, WHR and CI with lipid profiles, blood pressure, serum insulin and FBS levels was determined with Pearson’s correlation coefficient. One-way analysis of variance (ANOVA) was used for assessing associations between cardiovascular risk factors (diabetes, blood pressure and dyslipidaemia) and CI quartiles. Because there were few cases of diabetes, FBS level was used as a proxy of diabetes association with CI. All statistical analyses were performed by SPSS version 17 (SPSS Inc, Chicago, IL, USA). A *p*-value < 0.05 was considered significant.

## Results

Data of 150 menopausal women were completed and included in the final analysis in this study (response rate 91%). The mean age of the women was 56.8 years (± 7.64) with a range of 42 to 80 years. With regard to education, 85.2% of subjects were literate. The majority of participating women were housewives (86.7%) and 79.3% were married. Only eight women (5.4%) had diabetes, based on our definition. The physical and laboratory characteristics of the women are shown in Table 1. Table 2 illustrates the correlation matrix of the variables.

**Table 1.** Physical And Laboratory Characteristics Of The Study Population

*Variables*	*Mean*	*SD*	*Min*	*Max*
Weight (kg)	64.58	8.10	45.00	84.00
Height (cm)	156.88	6.19	142.00	178.00
BMI (kg/m^2^)	26.11	2.93	18.50	36.00
Waist circumference (cm)	87.18	9.07	67.00	107.00
Hip circumference (cm)	103.11	6.80	88.00	122.00
Waist–hip ratio	0.84	0.07	0.68	1.09
Systolic blood pressure (mmHg)	11.59	1.46	9.00	19.00
Diastolic blood pressure (mmHg)	7.49	0.96	6.00	11.00
Fasting blood sugar (mg/dl)	97.14	43.26	70.00	380.00
Plasma insulin level	8.19	4.26	1.10	24.40
Total cholesterol (mg/dl)	193.14	45.38	110.00	350.00
Triglycerides (mg/dl)	215.02	105.22	70.00	620.00
LDL-C (mg/dl)	111.81	41.93	21.30	260.00
HDL-C (mg/dl)	38.84	6.93	26.00	60.00
Fat mass	43.15	78.69	20.70	69.99
Conicity index	1.24	0.09	1.04	1.50

BMI: body mass index, LDL-C: low-density lipoprotein cholesterol, HDL-C: high-density lipoprotein cholesterol; SD: standard deviation.

**Table 2.** Correlation Matrix With Pearson Correlation Coefficient Of Measured Variables

	*BMI*	*WC*	*WHR*	*SBP*	*DBP*	*FBS*	*PIL*	*TC*	*TG*	*HDL-C*	*LDL-C*	*FM*	*CI*
BMI	–	0.67*	0.33*	0.21*	0.10	–0.06	–0.10	–0.11	–0.06	0.01	–0.04		0.31*
WC	–	–	–	0.26*	0.16*	–0.15	–0.08	–0.06	–0.12	–0.07	–0.04		0.86*
WHR	–	–	–	0.29*	0.10	–0.15	–0.03	–0.00	–0.15	–0.03	0.03	–0.00	0.84*
SBP	–	–		–	–	–0.05	–0.22*	0.20*	0.00	0.13	0.16*		0.22*
DBP	–	–		–	–	0.01	–0.15	0.10	0.04	0.11	0.04		0.08
FBS	–	–		–		–	0.23*	0.41*	0.28*	0.30*	0.29*		–0.16*
PIL						–	–	–0.06	0.29*	–0.09	–0.13		–0.07
TC	–	–	–	–	–	–	–	–	0.28*	0.15	0.84*		–0.06
TG	–	–	–	–	–	–	–	–	–	0.03	–0.06		–0.17*
LDL-C	–	–	–	–	–	–	–	–	–	–	–		–0.00
HDL-C	–	–	–	–	–	–	–	–	–	–	–		–0.07
FM													

BMI: body mass index, WC: waist circumference, WHR: waist–hip ratio, SBP: systolic blood pressure, DBP: diastolic blood pressure, FBS: fasting blood sugar, PIL: plasma insulin level, TC: total cholesterol, TG: triglycerides, LDL-C: low-density lipoprotein cholesterol, HDL-C: high-density lipoprotein cholesterol, FM: fat mass. *p < 0.05.

Results show that BMI was positively and significantly associated with SBP (*r* = 0.21; *p* = 0.009). WC was positively and significantly correlated with SBP (*r* = 0.26; *p* = 0.02) and DBP (*r* = 0.16; *p* = 0.05). WHR was positively and significantly associated with SBP (*r* = 0.29; *p* = 0.001). CI had a positive correlation with SBP (*r* = 0.22; *p* = 0.009). The association of CI with FBS (*r* = –0.16) and triglycerides (*r* = –0.17) was weak and negatively significant.

Because of the small number of subjects with diabetes in this study, the association between anthropometric measures and this risk factor for cardiovascular disease was not assessed. Table 3 shows the risk factor for cardiovascular disease and anthropometric measures according to quartiles of CI. This table shows that age and WC were associated with CI quartiles at the 0.05 significance level. The correlation of CI quartiles with SBP and weight were at the 0.01 significance level.

**Table 3.** Measured Variables (Mean And SD) Of Study Subjects According To Quartiles Of CI

	*CI*			
*Variables*	*1st Q < 1.18 (n = 37)*	*2nd Q 1.18 > 1.23 (n = 38)*	*3rs Q 1.23 < 1.31 (n = 38)*	*4th Q ≥ 1.31 (n = 37)*
Age*	55.37 (7.33)	55.76 (6.56)	56.29 (5.40)	60.18 (9.87)
Weight (kg)**	61.67 (7.56)	64.92 (8.82)	65.78 (7.03)	66.10 (8.24)
Height (cm)	157.62 (5.27)	157.26 (5.55)	156.72 (6.23)	156.02 (7.65)
SBP (mmHg)**	11.13 (1.31)	11.69 (1.70)	11.51 (1.36)	12.01 (1.37)
DBP (mmHg)	7.35 (1.06)	7.68 (0.98)	7.24 (0.98)	7.67 (0.74)
Waist (cm)*	77.29 (6.24)	84.39 (5.13)	89.75 (4.86)	97.43 (5.25)
FBS (mg/dl)	109.81 (69.33)	90.71 (24.57)	101.78 (42.76)	86.89 (11.99)
TC (mg/dl)	200.02 (49.44)	192.00 (45.57)	185.64 (43.53)	192.70 (41.93)
TG (mg/dl)	251.78 (135.79)	202.81 (87.87)	197.48 (98.54)	201.78 (80.97)
HDL-C (mg/dl)	40.45 (7.29)	38.63 (7.95)	37.27 (6.14)	39.00 (6.13)
LDL-C (mg/dl)	113.40 (43.12)	112.42 (45.90)	105.62 (37.53)	114.20 (41.25)

SBP: systolic blood pressure, DBP: diastolic blood pressure, FBS: fasting blood sugar, TC: total cholesterol, TG: triglycerides, LDL-C: low-density lipoprotein cholesterol, HDL-C: high-density lipoprotein cholesterol. *p < 0.05, **p < 0.01.

## Discussion

In this cross-sectional study, 165 postmenopausal women were randomly selected from the Health and Treatment Centre and Endocrine and Metabolism Research Centre of Firoozgar Hospital of Tehran University of Medical Sciences. Correlations of BMI, WC and CI with cardiovascular risk factors (hypertension, serum LDL-C and HDL-C, glucose and insulin levels) were assessed. WC had a significant correlation with SBP and DBP. BMI had a significant correlation with SBP only. CI had a significant correlation with SBP.

The findings of our study was similar to those of Taratchuk and co-workers.^13^ They reported that WC and CI were superior to BMI for identifying visceral adiposity, metabolic disorders and cardiovascular risk factors.^13^

Alemedia recommended a cut-off point of 86 cm for WC, 0.87 for WHR and 1.25 for CI as indicators of increased occurrence of cardiovascular risk factors, despite the lack of consensus between studies.^14^ Other studies gave 1.18 as the best cut-off point for CI. Almeida reported that CI had the highest sensitivity and specificity for predicting the occurrence of cardiovascular risk factors.^14^ In our study, the postmenopausal women had a mean WC of 87.1 ± 9 cm, WHR of 0.85 ± 0.07 and CI of 1.24 ± 0.9, which verified the use of central obesity to indicate a high probability of the occurrence of cardiovascular risk factors in these participants.

Zhou *et al*. reported that BMI, WC and CI had an association with blood pressure but WC in men and BMI in women had the highest association with blood pressure.^15^ In our study, only BMI had a significant association with SBP. In comparison to our study, Zhou’s study was done on a larger population (29 179 vs 150 participants). Zhou indicated that visceral obesity, measured by WC or WHR was more closely associated with blood pressure and/or the presence of hypertension than overall obesity, measured by BMI. Furthermore, the linear regression coefficient for each obesity measurement with continuous SBP or DBP was substantially greater in men than in women, suggesting a greater male responsiveness of blood pressure to a gain than weight or abdominal deposition.^15^

Dalton reported a higher association of BMI with blood pressure and LDL-C and HDL-C levels compared to WC. However, he had more participants compared to our study (11 247 vs 150 persons) and he did not make use of conicity index.^8^ Neufeld reported the best cut-off points for pre-diabetes status as 27.8 kg/m^2^ for BMI, 89.8 cm for WC and 1.28 for CI. In his study, BMI had more sensitivity and specificity with pre-hydration and pre-diabetes compared to WC and CI, but his study was carried out on under 35-year-old pre-menopausal women.^16^

The results of the studies by Ghosh, and Sanchez Viveros *et al*. in postmenopausal and elderly subjects, respectively, was consistent with our study. They reported that CI had a higher association with type 2 diabetes compared to BMI and WC.^17^,^18^ On the other hand, Ghosh indicated a significant difference between central obesity and fat-free mass among normotensive and hypertensive subjects, although their level of obesity was similar.^17^ Hypertensive individuals had significantly enhanced levels of central body fat distribution, which was consistent with the findings of our study.

A few studies reported that waist-to-height ratio and sagital abdominal diameter had the highest association with serum lipoprotein cholesterol levels and blood pressure.^19^,^20^ However we did not measure these two parameter but suggest that future studies do so.

Our study had some limitations. The most important was the small sample size, resulting in too few diabetic patients. This was due to difficulties in getting women to participate in the study. However, a sample size of 100 should be enough to assess the correlation between variables within one group.

Another limitation was the nature of cross-sectional studies, which are not able to determine temporality. Therefore we could not identify whether the risk factors of cardiovascular disease preceded increased adiposity, or increased adiposity was the result of these risk factors (dyslipidaemia, diabetes and hypertension). We suggest future studies with prospective designs are necessary to identify this enigma.

## Conclusion

We showed a significant association of WC with SBP and DBP, which are important risk factors for cardiovascular disease. We also indicated BMI could be an important determining factor of SBP. Although we showed a significant association between CI and SBP, it did not have enough power and more investigations are necessary.

## References

[1] Denke MA, Sempose CT, Grundy SM (1993). Excess body weight: An under-recognized contributor to high blood cholesterol levels in white American men.. Arch Intern Med.

[2] Kannel WB, D’Agostino RB, Cobb JL (1996). Effects of weight on cardiovascular disease.. Am J Clin Nutr.

[3] Eckel RH, Krauss RM (1998). American Heart Association Call to Action: Obesity as a major risk factor for coronary heart disease.. Circulation.

[4] Mirmiran P, Esmaillzadeh A, Azizi F (2004). Detection of cardiovascular risk factors by anthropometric measures in Tehranian adults: receiver operating characteristic (ROC) curve analysis.. Eur J Clin Nutr.

[5] Wang Y, Rimm EB, Stampfer MJ, Willett WC, Hu FB (2005). Comparison of abdominal adiposity and overall obesity in predicting risk of type 2 diabetes among men.. Am J Clin Nutr.

[6] Colditz G, Willett W, Rotnitzky A, manson J (1995). Weight gain as a risk factor for clinical diabetes mellitus in women.. Ann Intern Med.

[7] Ho SC, Chen YM, Woo JL, Leung SS, Lam TH, Janus ED (2001). Association between simple anthropometric indices and cardiovascular risk factors.. Int J Obes Relat Metab Disord.

[8] Dalton M, Cameron AJ, Zimmet PZ, Shaw JE, Jolley D, Dunstan DW (2003). Waist circumference, waist-hip ratio and body mass index and their correlation with cardiovascular disease risk factors in Australian adults.. J Intern Med.

[9] Huang B, Rodreiguez BL, Burchfiel CM, Chyou PH, Curb JD, Sharp DS (1997). Association of adiposity with prevalent coronary heart disease among elderly men: The Honolulu heart program.. Int J Obes.

[10] Lemieux S, Prudhomme D, Bouchard C, Tremblay A, Després JP (1996). A single threshold value of waist girth identifies normal-weight and overweight subjects with excess visceral adipose tissue.. Am J Clin Nutr.

[11] Valdez R, Seidell JC, Ahn YI, Weiss KM (1993). A new index of abdominal adiposity as an indicator of risk for cardiovascular disease. A crosspopulation study.. Int J Obese.

[12] Kim KS, Owen WL, Williams D, Adams-Campbell LL (2000). A comparison between BMI and conicity index on predicting coronary heart disease: The Framingham heart study.. AEP.

[13] Tarastchuk JC, Guerios EE, Bueno R, Andrade PM, Nercolini DC, Ferraz JG (2008). Obesity and coronary intervention: should we continue to use body mass index as a risk factor?. Arq Bras Cardiol.

[14] Almeida RT, Alemida MM, Araujo TM (2009). Abdominal obesity and cardiovascular risk: performance of anthropometric indexes in women.. Arq Bras Cardio.

[15] Zhou Z, Hu D, Chen J (2008). Associating between obesity indices and blood pressure or hypertension: which index is the best?. Public Health Nutr.

[16] Neufeld LM, Jones-Smith JC, Garcia R, Fernald LC (2008). Anthropometric predictors for the risk of chronic disease in non-diabetic, non- hypertensive young Mexican women.. Public Health Nutr.

[17] Ghosh A (2009). Obesity measures metabolic profiles and dietary fatty acids in lean and obese postmenopausal diabetic Asian Indian women.. Anthropol Anz.

[18] Sanchez-Viveros S, Barquera S, Medina-Solis CE, Velazquez-Alva MC, Valdez R (2008). Association between diabetes mellitus and hypertension with anthropometric indicators in older adults: results of the Mexican Health survey.. J Nutr Health Aging.

[19] Kotona-Durekovic A, Stokic E (2006). The significance of some anthropometric parameters and parameters ensuing from them in assessing cardiovascular risk in type 2 diabetes.. Med Preg l..

[20] Gustat J, Elkasabany A, Srinivassan S, Berenson GS (2000). Relation of abdominal height to cardiovascular risk factors in young adults.. Am J Epidemiol.

